# Dispelling the ethical apprehensions surrounding same day cataract consent

**DOI:** 10.1038/s41433-022-02348-0

**Published:** 2022-12-12

**Authors:** Rosina Zakri, Hasan Naveed, Robert Hill, Rashid Zia

**Affiliations:** 1 Guys and St Thomas’ NHS Foundation Trust, London, UK; 2 Frimley Health NHS Foundation Trust, Frimley, UK; 3 University Hospitals Sussex Foundation Trust, Worthing, UK; 4 Herne Bay Ophthalmology Services, Herne Bay, UK

**Keywords:** Lens diseases, Public health

The suitability of the same day cataract surgery as a viable option for delivering service has been recently suggested in response to growing surgical demand in every region of the UK. Examples of successful models have been published from Bristol (Hughs et al., 2001) [1] Wales (Evans et al., 2004) [2] and most recently Scotland (Dhillon et al., 2021) [3], addressing each aspect of the service pathway. Whilst the issues surrounding adequate triage and theatre utilisation are discussed, the ethical dilemma of ‘short time frame’ between consent and surgery remains a hot topic of debate.

Despite there being no legal length of time between obtaining consent and performing a procedure, the Department of Health clearly states that consent cannot be taken under duress [4]. It is therefore stipulated by Kerns J. in the Fitzpatrick case (2008), risks of surgery should not be provided to the patient at the ‘eleventh hour’ and thus a ‘cooling off’ period may be required [5]. Although the same day procedures for many retinal conditions, such as intravitreal injections and laser, are commonly recognised as patient centric, there is still opposition to similar benefits when it comes to cataract surgery.

Consent is defined by Brazier as the process in which intellectually competent patients accept or reject the treatment proposed to them, based on the autonomy of sovereignty over their own body [6]. Under Common Law patients have the right to be involved in decisions about their treatment, including those with (and to an extent without) capacity. The law states that for consent to be valid, patients should be informed of potential risks and reasonable alternatives to recommended treatment. If this is not done in an adequate manner (as per the Bolam Test) [7], healthcare professionals may be liable to the tort of battery, or even criminal assault.

In 2020 the General Medical Council updated its guidance on informed consent in England. They state that discussions should be tailored to each individual patient and be guided by what is important to them personally (as per the landmark Montgomery ruling (2015)) [8].

Based on this awareness, along with the issues already raised, Herne Bay Ophthalmology Services was set up to provide a ‘one-stop’ cataract service in Kent and has been offering the same day cataract surgery for the last decade. Patients from the Kent region attend and are able to sign for consent on the day of surgery. However, at our facilities discussions around cataract surgery begin by the referring optician at time of diagnosis. Once triaged to our practice, the service ensures that patients receive written information 2 weeks prior to the appointment to continue the consent dialogue. Information includes risks of surgery, the procedure itself and post-procedure advice. They also receive a phone call from one of the staff to ensure they understand the information provided, which may itself initiate formal remote consultation if required.

Patients arrive at their booked appointment already armed with background, knowledge and having considered the options, are often eager to proceed to surgery. Patients are then seen in a focussed one-stop clinic by a senior doctor to assess suitability and risk factors before they are subsequently consented in this setting. Those that require more time to consider their options are booked as per conventional two-stop services, and those who wish to take advantage of the unique same day option can also proceed.

Service reviews of this model of care delivery have deemed it to be sustainable and financially viable as services are rarely underutilised. A recent survey concluded that 83% of patients using our service opted for the same day option, with 100% top rating of ‘very good’ post procedure. In fact, in our experience, patients are eager to arrive early to avail the opportunity of being listed for surgery on the day before the list becomes full.

We would therefore suggest that so long as the process keeps the patient at the centre of its care and appropriate step-wise communication is maintained, consent can be taken ethically and with validity for surgery on the day of signing the consent form. We would suggest that an understanding of moral and legal aspects of consent has allowed us to adapt conventional services to meet the growing demands of healthcare whilst facing the challenges of consent, triage and theatre utilisation head on. We encourage other regions to consider this evidence as one potential solution to be used alongside national guidelines to tackle the backlog of services in Ophthalmology, such as age-related cataracts.

## References

[1] Hughes E, Forrest F, Diamond J (2001). ‘One-stop’ cataract surgery: The Bristol Eye Hospital experience 1997-1999. Eye.

[2] Evans SL, Saunders D, Haslett R (2004). One-stop cataract clinics: feasible but flawed?. Eye.

[3] Dhillon N, Ghazal D, Harcourt J, Kumarasamy M. A proposed redesign of elective cataract services in Scotland – pilot project. Eye. 2022;36:2116–21. 10.1038/s41433-021-01810-9. 10.1038/s41433-021-01810-9 PMC9581890 34686778

[4] Department of Health and Social Care. Reference guide to consent for examination or treatment. 2nd ed. 2009. https://www.gov.uk/government/publications/reference-guide-to-consent-for-examination-or-treatment-second-edition. Accessed 13 Nov 2022.

[5] Getting informed consent from your patients. 2018. https://kennedyslaw.com/media/3086/kennedys_gettinginformedconsentfromyourpatients.pdf.

[6] McLean S. First do no harm: law, ethics and healthcare. Abingdon, Oxon: Routledge; 2016.

[7] Lawteacher.net. The Bolam test in clinical negligence. 2019. https://www.lawteacher.net/free-law-essays/medical-law/bolam-test-clinical-negligence.php.

[8] Guidance on professional standards and ethics for doctors decision making and consent. (n.d.). https://www.gmc-uk.org/-/media/documents/updated-decision-making-and-consent-guidance_pdf-84160128.pdf.

